# Dynamic performance verification of the Rędziński Bridge using portable camera-based vibration monitoring systems

**DOI:** 10.1007/s43452-022-00582-7

**Published:** 2022-12-02

**Authors:** Mateusz Bocian, Nikolaos Nikitas, Maksat Kalybek, Mieszko Kużawa, Paweł Hawryszków, Jan Bień, Jerzy Onysyk, Jan Biliszczuk

**Affiliations:** 1 Department of Roads, Bridges, Railways and Airports, Faculty of Civil Engineering, Wrocław University of Science and Technology, Wybrzeże Stanisława Wyspiańskiego 27, 50-370 Wrocław, Poland; 2 School of Civil Engineering, University of Leeds, Woodhouse Lane, Leeds, LS2 9DY UK; 3 School of Engineering, University of Leicester, University Road, Leicester, LE1 7RH UK; 4 Faculty of Engineering Science, University College London, Gower Street, London, WC1E 6BT UK

**Keywords:** Remote sensing, Computer vision, Modal damping, Long-span bridges, Operational modal analysis

## Abstract

The assessment of dynamic performance of large-scale bridges typically relies on the deployment of wired instrumentation systems requiring direct contact with the tested structures. This can obstruct their operation and create unnecessary risks to the involved personnel and equipment. These problems can be readily avoided by using non-contact instrumentation systems. However, the cost of off-the-shelf commercial products often prevents their wide adoption in engineering practice. To this end, the dynamic performance of the biggest one-pylon cable-stayed bridge in Poland is investigated based on data from a consumer-grade digital camera and open access image-processing algorithms. The quality of these data is benchmarked against data obtained from conventional wired accelerometers and a high-end commercial optical motion capture system. Operational modal analysis is conducted to extract modal damping, which has a potential to serve as an indicator of structural health. The dynamic properties of the bridge are evaluated against the results obtained during a proof loading exercise undertaken prior to the bridge opening. It is shown that a vibration monitoring system based on consumer-grade digital camera can indeed provide an economically viable alternative to monitoring the complex time-evolving dynamic behaviour patterns of large-scale bridges.

## Introduction

Although camera-based motion capture systems (MCS) have been extensively studied in the last few decades [1–7], their applications in monitoring large-scale structures are rarely reported. Instead, the vast majority of studies is concerned with the validation and performance assessment of increasingly sophisticated MCS and tracking algorithms by means of experimental testing of scaled-down simplified structural models in a laboratory environment [8, 9]. Although such tests serve their purpose well and are entirely justified as they enable the influence of uncontrolled factors to be eliminated, or at least reduced, the end goal in the development of camera-based MCS is their in situ deployment on full-scale structures.

When it comes to investigating in situ bridges, the main focus is on footbridges, typically spanning several dozen metres [4]. Only few studies employing camera-based MCS were conducted on bridges having spans of several hundred metres. These studies involved the Humber Bridge [10, 11] and Second Severn Crossing [12] in the UK, the Vincent Thomas Bridge [13], Manhattan Bridge [14] and Bronx-Whitestone Bridge [15] in the USA, the Guadiana Bridge in Portugal [16], the Gwangan Bridge [17] and Busan-Geoje Bridge [18] in South Korea, the Tsing Ma Bridge in Hong Kong [19] and the Chi-Lu Bridge in Taiwan [20]. However, it can be argued that the benefits of using camera-based MCS become particularly tangible in the case of large bridges. This is because these bridges are often accessible to vehicular traffic only, while the pedestrian traffic is disallowed, therefore costly arrangements must be made for the ad hoc vibration monitoring system requiring direct contact with the structure to be safely accommodated.

The direct output of measurements of in situ bridge behaviour using camera-based MCS is displacement. This is in some cases more useful than acceleration as it can be readily adopted for comparison against numerical models of structures and it forms the basis for the derivation of strain [21], hence it can inform of the stress state within the structure. However, to the best of authors’ knowledge, camera-based MCS have never been used for the identification of modal damping from large-scale bridges in situ [15]. The knowledge of this parameter is a prerequisite for calibrating numerical models of bridges for the purpose of analysing their response to various types of dynamic loading. Instead, a common practice is to perform this task from measurements taken with wired instrumentation systems requiring direct contact with the structure during dedicated proof loading tests [22]. Although necessary at the structure’s commissioning stage, these tests are typically too expensive and time-consuming to be regularly repeated. Performing regular modal identification tests could, however, enable valuable information to be collected on structural health, which in turn enables effective management and maintenance of bridges [23].

In this context, damping has been suggested to serve as an indicator of structural health. Numerous empirical studies on relatively simple structural elements have shown damping to be more sensitive to the level of structural damage than modal frequencies and mode shapes, alongside their spatial derivatives [24]. For composite structures, e.g. these made of concrete and steel, such as the Rędziński Bridge, the structural damage has been typically associated with increased damping due to the increased friction at the crack and/or materials’ debonding interfaces [25]. While the applicability of this methodology in the case of full-scale bridges requires further investigation, its potential feasibility is an attractive prospect.

Considering the abovementioned rationale, the aim of this study was to investigate the feasibility of using camera-based vibration monitoring systems for the determination of modal damping on large-scale bridges in situ. The dynamic behaviour of the Rędziński Bridge in Poland was measured by means of four monitoring systems on the 23^rd^ of November, 2020. These included conventional wired accelerometers, a commercial optical MCS and two MCS relying on a consumer-grade digital camera (CGC) with two open source motion tracking algorithms. For comparison, another set of measurements taken on the 16^th^ of August, 2011 was used to evaluate the bridge’s dynamic characteristics prior to its opening. The rest of the paper is organised as follows. The tested structure, deployed instrumentation systems, experimental protocol and data processing are introduced in Sect. 2. Section 3 presents and discusses results from a comparative study on the dynamic bridge response characteristics in terms of the recovered kinematics and modal damping. To the best of the authors’ knowledge, the level of detail achieved in the analyses and findings summarised in the closing Sect. 4 surpass any previous similar study on this topic.

## Materials and methods

The dynamic response of the Rędziński Bridge under ambient loading, mainly vehicular traffic, was measured on the 23rd of November, 2020. This section provides a brief description of the bridge, instrumentation systems, experimental protocols and data processing. It also provides information on the proof loading tests conducted on the 16th of August, 2011, albeit in lesser detail since most results of those tests have already been reported in [26].

### Rędziński Bridge

**Fig. 1:**
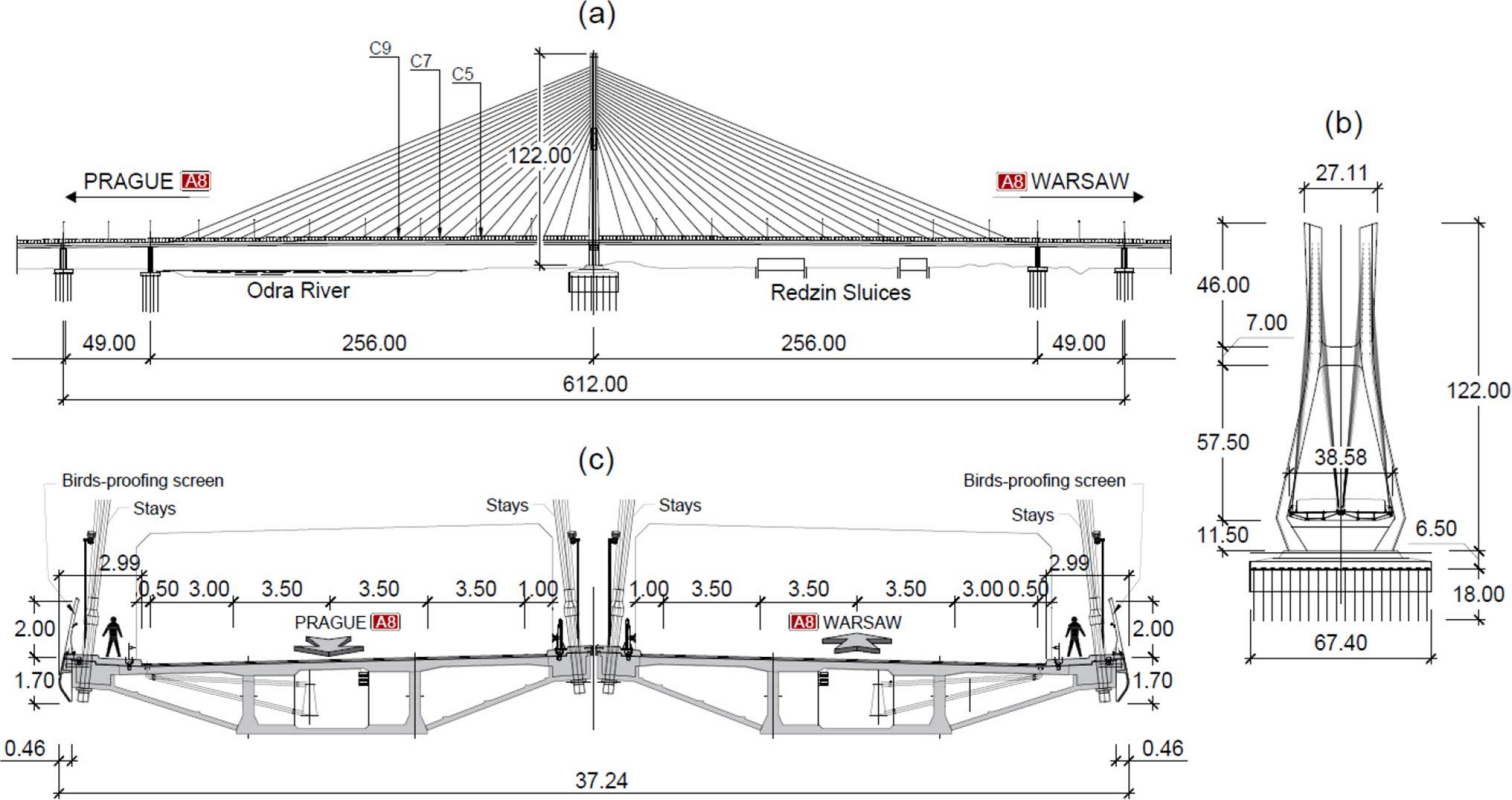
**a** Elevation of the central section of the Rędziński Bridge, **b** front view of the pylon, and **c** cross section through the deck [curtesy of Mosty Wrocław – engineering consultancy and bridge designer; www.mostywroclaw.com.pl]. C5, C7 and C9 depicted in (**a**) denote measurement points. All dimensions are in metres

The Rędziński Bridge is the biggest one-pylon cable-stayed bridge in Poland. Inaugurated in September 2011, it forms a part of the northern bypass of Wrocław, the capital of Lower Silesia voivodeship. The bridge is 1742 m long and consists of the central 612-m-long cable-stayed section with two auxiliary supports, and outer multi-span flyovers. The bridge’s pylon and piers sit on bored and prefabricated piles, respectively [27]. The 122-m-tall steel-core concrete pylon has two kinks in each of its two legs, such that the legs’ separation increases from the ground level towards the level of the decks supported by the lower cross-beam, and then decreases up to the point of the upper cross-beam. The upper cross-beam marks the beginning of the stay cables anchorage section of the legs. The bridge carries 3 lanes of traffic, a hard shoulder/service lane and a service pathway on each of the two mutually independent concrete continuous box girder decks, each supported by one of the pylon’s legs. Each of the four main sections extending out from the pylon is 256 m long and supported by 20 pairs of cable stays. The elevation of the cable-stayed section of the bridge and cross sections through the pylon and the deck are shown in Fig. 1. Due to environmental factors, i.e. the bridge being located over the Odra River and at the crossway of birds’ corridors, a 2-m-tall screen is installed along the outermost edges of the decks, providing a physical barrier guarding vehicles against birds’ strikes and vice versa. A more detailed description of the bridge can be found in [28].

**Fig. 2:**
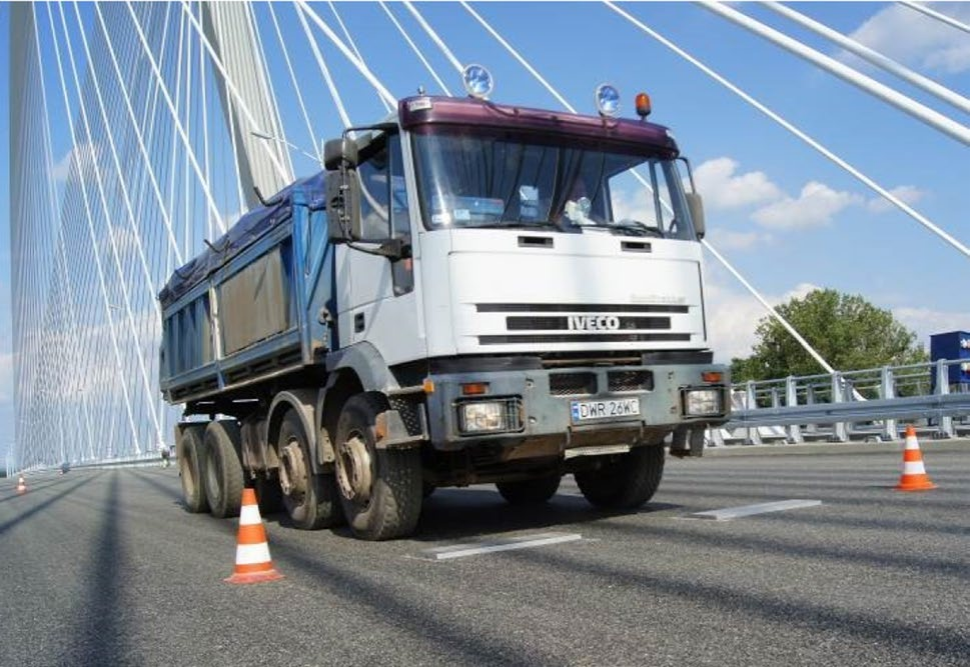
A lorry driven over a bump during a proof loading test on the Rędziński Bridge in August 2011, after [29]

The dynamic behaviour of the bridge was investigated during a proof loading test on the 16^th^ of August, 2011 [26]. Several dominant vibration frequencies below 1 Hz were identified from measurements of the vertical bridge response using accelerometers and linear variable differential transducers (LVDTs). These frequencies and associated deflection shapes were in line with the predictions of modal characteristics from numerical models of the bridge available at the time [26]. As part of the testing protocol, a loaded lorry was driven at various speeds across a 3-cm-high bump situated within the middle lane of the deck carrying traffic towards Warsaw to amplify its response, as shown in Fig. 2 (tests termed D3 as reported in [26]). Other performed tests involved single lorry passages over different lanes and at various speeds (tests termed D2 as reported in [26]), or side-by-side passages of multiple lorries (tests termed D1 as reported in [26]). The data collected during those tests were used herein to evaluate modal damping, reported in Sect. 3.4, which has not been previously attempted neither in [26] nor in any other studies on the Rędziński Bridge.

### Instrumentation

The instrumentation systems deployed on the 23^rd^ of November, 2020, included wired accelerometers, Imetrum – commercial optical MCS and two systems based on a consumer-grade camera (CGC). A light intensity meter was also deployed on site to track changes in ambient illumination. Basic specifications of the instrumentation systems used in the most recent experimental campaign, as provided by the manufacturers, are given in Table 1 and more details are given in the following sections. Table 1 also contains information on the instrumentation systems deployed during the proof loading tests undertaken on the 16^th^ of August, 2011, as reported in [26].

**Table 1:** Basic specifications of the instrumentation systems used during experimental campaigns

System	Sensor	Quantity	Maximum operational frequency and resolution	Distance relative to the measurement point of interest^a^
**23 November 2020**				
Accelerometry	ASC 4211LN-002 & HBM QuantumX	6 & 1	300 Hz at ± 2 g & 24 bit	Directly at C5, C7 and C9
Imetrum	Manta G-223 B NIR with Nikon 180 mm lens	1	50 fps at 2048 × 1088 pixels	Approximately 100 m
Consumer-grade camera (CGC)	Canon EOS 200 D with DIGIC 7 processor and 70–300 mm Canon lens	1	59.94 fps at 1920 × 1080 pixels	Approximately 100 m
Light intensity meter	Chauvin Arnoux CA1110	1	1 Hz	Approximately 100 m
**16 August 2011**				
Accelerometry	HBM B12/200 & HBM Spider8	8 & 1	100 Hz at ± 20 g & 16 bit	Directly between C7 and C8^b^
Linear variable differential transformers (LVDT)	HBM W50TS & HBM Spider8	8 & 1	100 Hz at ± 50 mm & 16 bit	Directly between C7 and C8^b^

^a^C*N* denotes a measurement point close to the location at which the *N*th cable, counting away from the pylon, is connected to the deck, as shown in Fig. 1 and Fig. 3

^b^C8 is located approximately midway between C7 and C9

#### Accelerometers

**Fig. 3:**
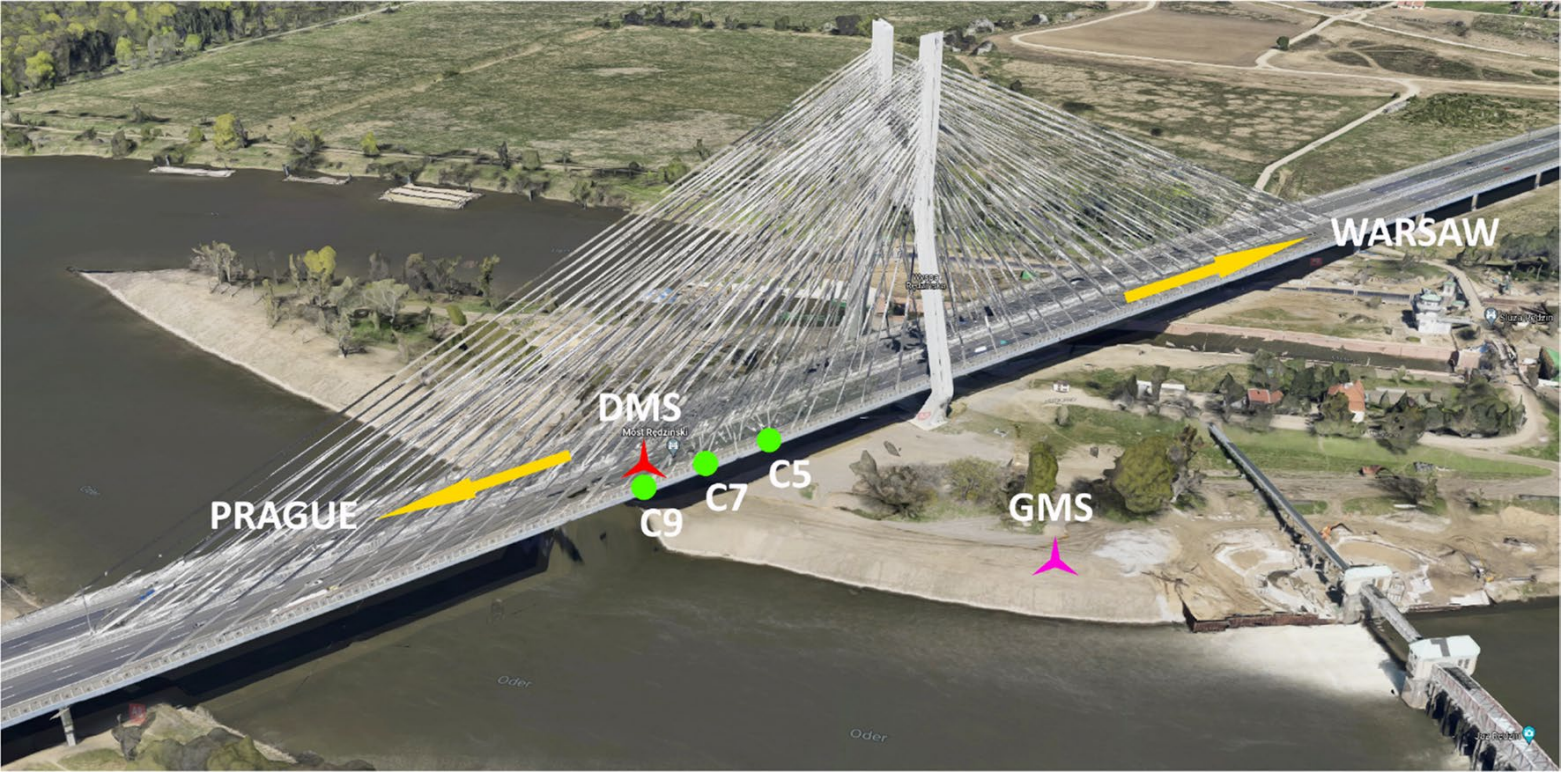
Layout of the testing site together with the location of the measurement points (green dots), deck measurement station (DMS; red star) and ground measurement station (GMS; pink star). Source: Google Maps

Acceleration of the deck was measured with six ASC 4211LN MEMS capacitive accelerometers with dynamic range of ± 2 g. Two accelerometers were installed at each of the measurement points, i.e. C5, C7 and C9, as shown in Fig. 1, along the outer edge of the southern deck, as shown in Fig. 3, to measure vertical and transverse bridge motion. The HBM QuantumX 24 bit data acquisition system with proprietary software was used to capture data from accelerometers, sampling at 300 Hz.

#### Imetrum

Imetrum is a commercial MCS consisting of an industrial-grade camera, dedicated PC and proprietary software (Video Gauge™). Originally developed for monitoring the Humber Bridge and Second Severn Crossing in the UK, Imetrum is currently one of the leading commercial suppliers of camera-based MCS. The motion tracking relies on a pattern recognition algorithm, enabling sub-millimetre precision to be achieved during typical application on road bridges [30].

#### Consumer-grade camera with motion tracking algorithms

A Canon 200D (24.2 MP) consumer-grade digital camera (CGC) equipped with a Canon 70–300 mm lens, set at the maximum zoom (i.e. 300 mm), with autofocus mode disabled was used to record full HD (1920 × 1080 pixels) videos of the bridge at 59.94 Hz. The camera was mounted on a tripod via a lens supporting bracket to ensure rigidity.

A custom-built video processing software package was used to extract the displacement signals from the Rędziński Bridge. The tools are based on two motion tracking algorithms commonly used in SHM, i.e. area-based template matching, hereafter referred to as template matching and denoted TM, and Lucas-Kanade sparse optical flow estimation, hereafter referred to as optical flow and denoted OF. The software was written in C +  + language using the Visual Studio 2017 programming environment, partly referring to the open source computer vision library (OpenCV; [31]) to enable camera calibration, specification of the transfer function between the 2D (planar) world coordinate system and the image coordinate system and tracking of specified regions of interest (ROI) within the video images.

The area-based template matching (TM) is a method based on sliding the template image selected as ROI across the video frame image and searching for the best match by calculating the similarity between the two [32]. Since the monitoring took place in the outdoor environment during a partially cloudy day, a normalized version of the correlation coefficient was used as a correlation criterion [31 – see p. 216], which is proven to be robust against light intensity fluctuations [33]. Once the areas matching the template images are located, their pixel coordinates are further refined to the subpixel level using the enhanced cross-correlation interpolation method [34] and then converted to the physical world coordinates.

The Lucas-Kanade sparse optical flow (OF) [31 – see p. 324] is a method that tracks sparse feature points (e.g. edges and corners) across consecutive images by estimating the flow (or motion) of these points caused by pixel intensity change. In this study, the feature points were first extracted from within the predetermined ROI using the Shi-Tomasi method [35]. Then, the optical flow at these points was estimated using the Lucas-Kanade algorithm [36]. The average pixel coordinates of tracked points for each ROI are then calculated and converted to the physical world coordinates.

### Testing protocol

The testing campaign was conducted in the morning and early afternoon on the 23rd of November, 2020. A ground measurement station (GMS) was set on the Wyspa Rędzińska (Rędzin Island) to the East of the bridge, as shown in Fig. 3. The camera-based MCS and the light meter were positioned approximately 100 m away from the measurement points located within the south-east section of the cable-stayed deck carrying traffic towards Warsaw. These points were chosen to enable all main modes having vertical motion component to be captured while ensuring the camera incidence angle remains as small as possible to minimise errors associated with the application of planar homography. The angle of incidence is defined herein as the angle between the centre of the camera sensor to the plane of interest within the captured image, here defined by the outermost face of the deck. For convenience and ease of identification, the measurement points were chosen to coincide with the locations at which the fifth, seventh and ninth cable stays, counting away from the pylon, are connected to the deck, hence they are referred to as C5, C7 and C9, respectively.

Five tests were conducted in total, each lasting approximately 10 min. A summary of the tests and their identifiers (i.e. ID) used hereafter are given in Table 2.

**Table 2:** Summary of the conducted tests

Test ID	Accelerometry	CGC	Imetrum	Approximate duration [mins]
T1	6 channels	1 camera pointing at C7	1 camera pointing at C7	10
T2	6 channels	1 camera pointing at C7	1 camera pointing at C7	10
T3	6 channels	1 camera pointing at C9	n/a	10
T4	6 channels	1 camera pointing at C9	n/a	10
T5	6 channels	1 camera pointing at C5	n/a	10

**Fig. 4:**
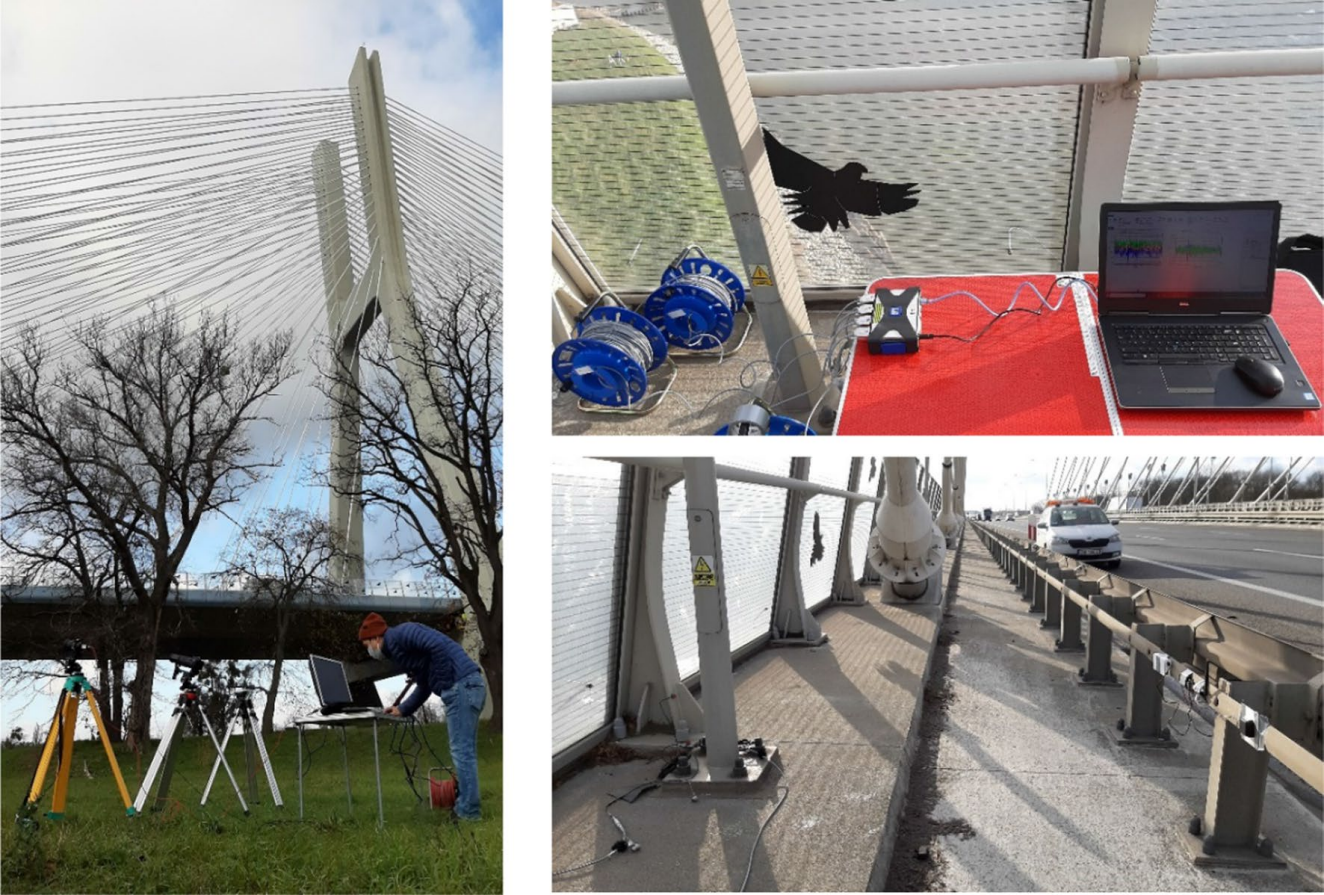
Equipment layout at the ground measurement station (GMS; left), the deck measurement station at C7 (DMS; top right), and wired accelerometers mounted at C9 (bottom right)

The CGC was adjusted such that the measurement points were, in turn, located in the middle of the recorded images; see Table 2. This was to address any potential lens edge distortion issues. The Imetrum, positioned in very close vicinity to CGC at GMS, was powered by a portable power generator set approximately 30 m away from the GMS. The initial attempts to conduct tests with Imetrum equipped with a 300 mm lens failed due to the image overexposure. Therefore, a 180 mm lens was used instead, but the same issue occurred during T3, T4 and T5, hence the only tests for which data from Imetrum are available are T1 and T2. Fluctuations in light intensity are known to affect the performance of optics-based vibration monitoring systems [15]. Therefore, a light meter (Chauvin Arnoux C.A 1110) was deployed next to the optical MCS to monitor the ambient lighting conditions locally. The GMS is shown on the left picture in Fig. 4.

A deck measurement station (DMS) was set within the service pathway of the south-east section of the cable-stayed deck carrying traffic towards Warsaw. A system acquiring data from three single-axis accelerometers mounted at each measurement point was set close to C9, as shown in the top right picture in Fig. 4.

Also shown on the bottom right picture in Fig. 4 is a safety car provided by the Polish General Directorate for National Roads and Motorways (Generalna Dyrekcja Dróg Krajowych i Autostrad, GDDKiA) securing the testing site on the bridge. This type of arrangement is usually enforced by the bridge operators and can significantly increase the cost of periplectic dynamic testing campaigns with instrumentation systems requiring direct contact with the structure.

**Fig. 5:**
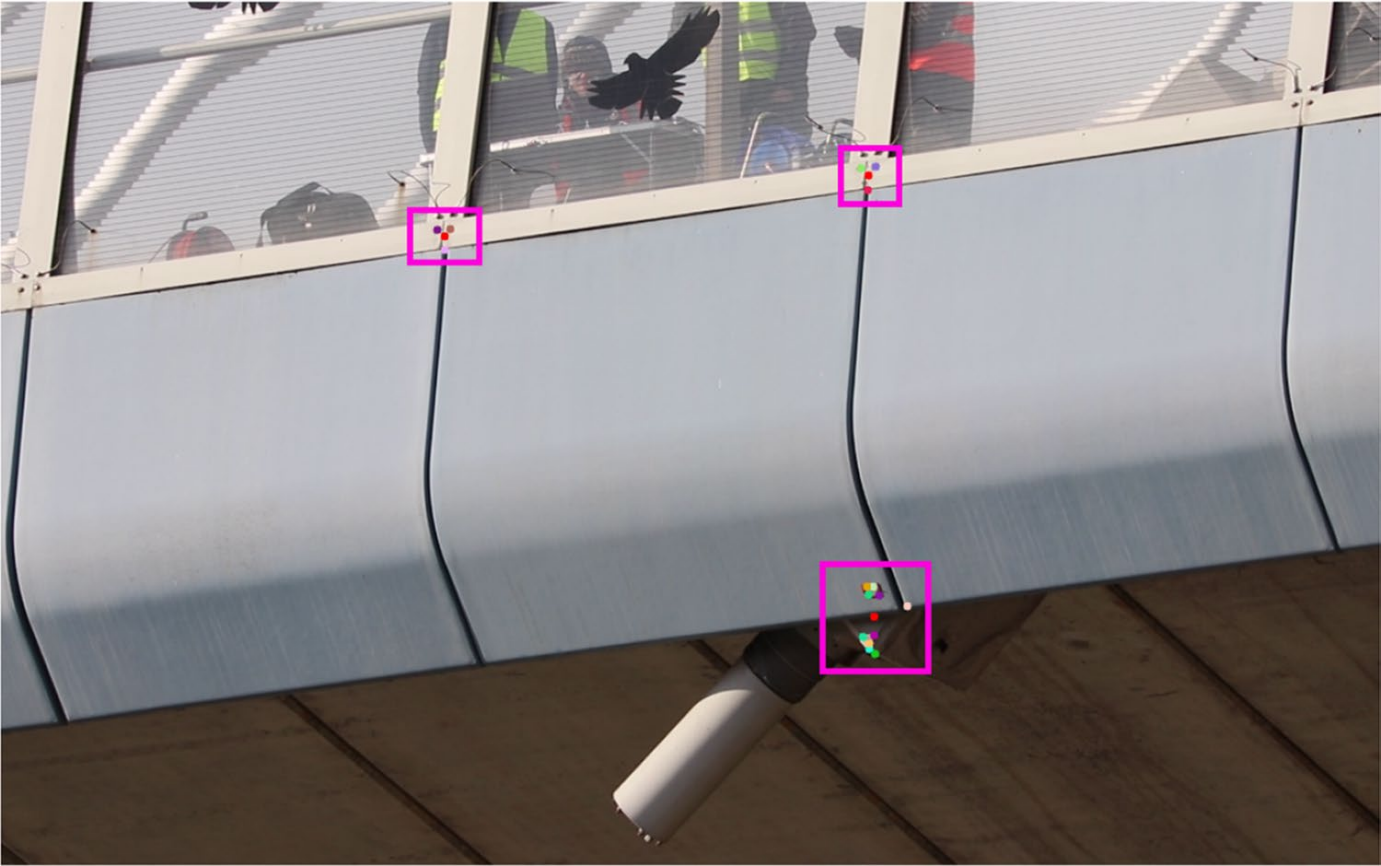
Camera view of C9 together with the ROI (pink rectangles) being tracked with the OF algorithm. The coloured dots within each ROI denote feature points consisting of one or several pixels having similar intensity

Because the outermost faces of the deck were not directly visible to the cameras due to the installed windshield, alternative measurement points had to be defined. These points were chosen at the top boundaries of the bottom section of the windshield, as shown in Fig. 5, to ensure their displacement is representative of that of the deck, since they coincide with the locations of the mounting brackets. Exemplar points captured with the optical flow algorithm are shown in Fig. 5. Only the data from one of the top outlined locations were used in the subsequent analysis to ensure match with the data from accelerometry.

### Data processing

Frequency-domain decomposition (FDD) [37] was used to extract modal damping for the major part of the analysis. The method was applied in a semi-automatic manner, prescribing the frequencies where modal damping ratios were to be identified. To check the impact of the chosen damping extraction method on the results, a variant of stochastic subspace identification (SSI) [38] was also used. A simple peak picking (PP) approach was also considered for the identification of dominant response frequencies, as in the previous study [26], to keep comparisons consistent.

## Results and discussion

### Dynamic bridge response

**Fig. 6:**
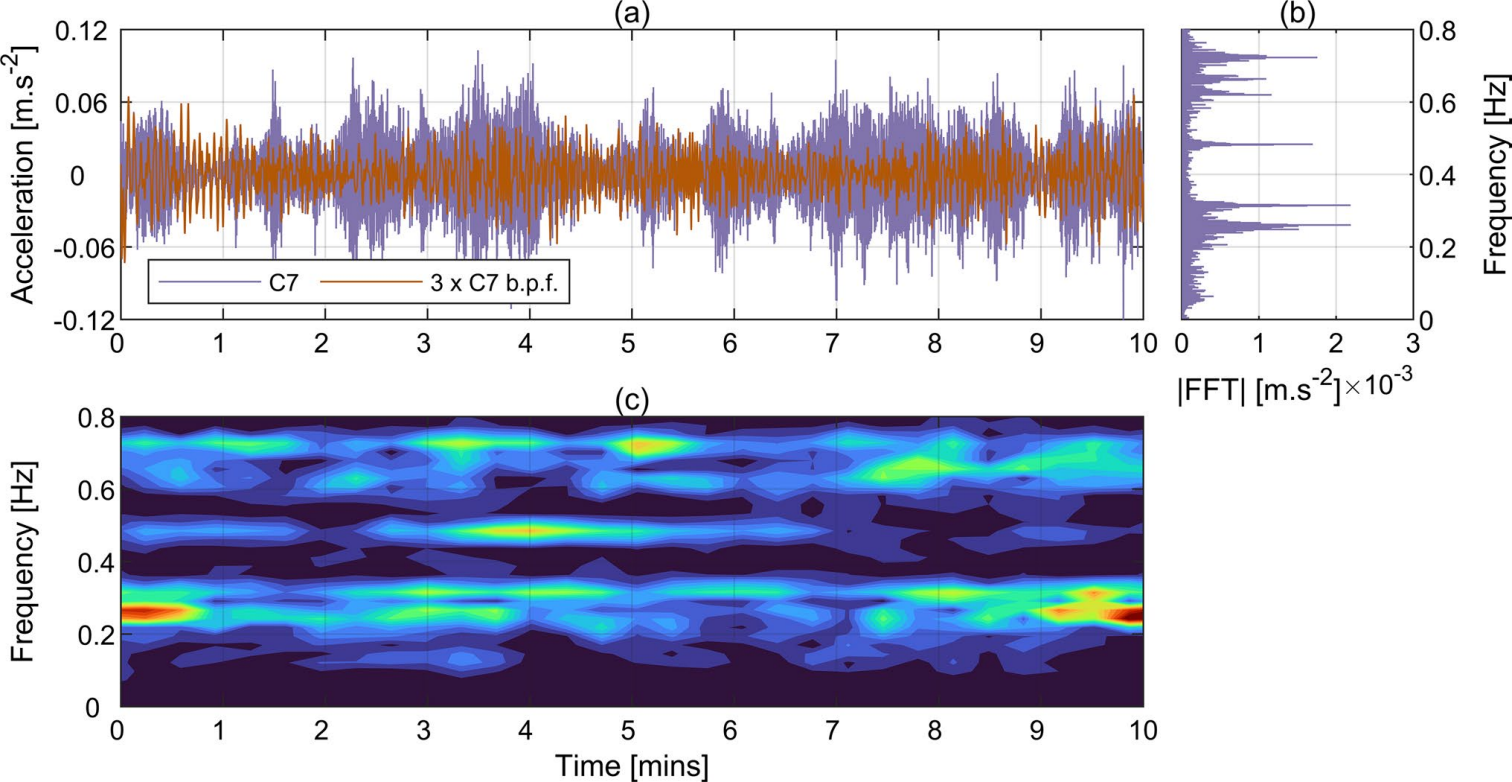
Acceleration measured during T2 at C7. **a** Raw time history and time history after the application of band-pass filter within the range from 0.1 Hz and 0.9 Hz with threefold magnification, **b** power-preserving FFT magnitude of raw acceleration, **c** Fourier spectrogram of the band-pass filtered acceleration

The dynamic bridge response captured with CGC was first evaluated against that from accelerometry and Imetrum. Overall, the match between the results from various instrumentation systems was consistent between the tests, bearing in mind the unavailability of Imetrum data for T3, T4 and T5 as discussed in Sect. 2.3. However, since the most complete set of data comes from T2, the results from that test are used herein to exemplify the main findings. The results from CGC TM are not presented, as they were found very similar to those from CGC OF. Figure 6a presents the raw acceleration measured at C7 during T2. The corresponding power-preserving magnitude of fast Fourier transform (FFT) of that signal is shown in Fig. 6b. Six dominant harmonic components can be clearly seen in the spectrum, corresponding to the six expected modes [26]. The acceleration amplitudes for these components reach a maximum at approximately 0.0022 m.s^−2^, and they comprise relatively little part of the original signal. To reveal this feature, the raw acceleration signal was filtered using the 4th order two-way Butterworth band-pass filter with cut off frequencies at 0.1 and 0.9 Hz. The resultant signal, magnified threefold for better visibility, is shown in Fig. 6a. Figure 6c presents a Fourier spectrogram of that filtered acceleration signal, indicating various frequency components were excited intermittently during T2.

**Fig. 7:**
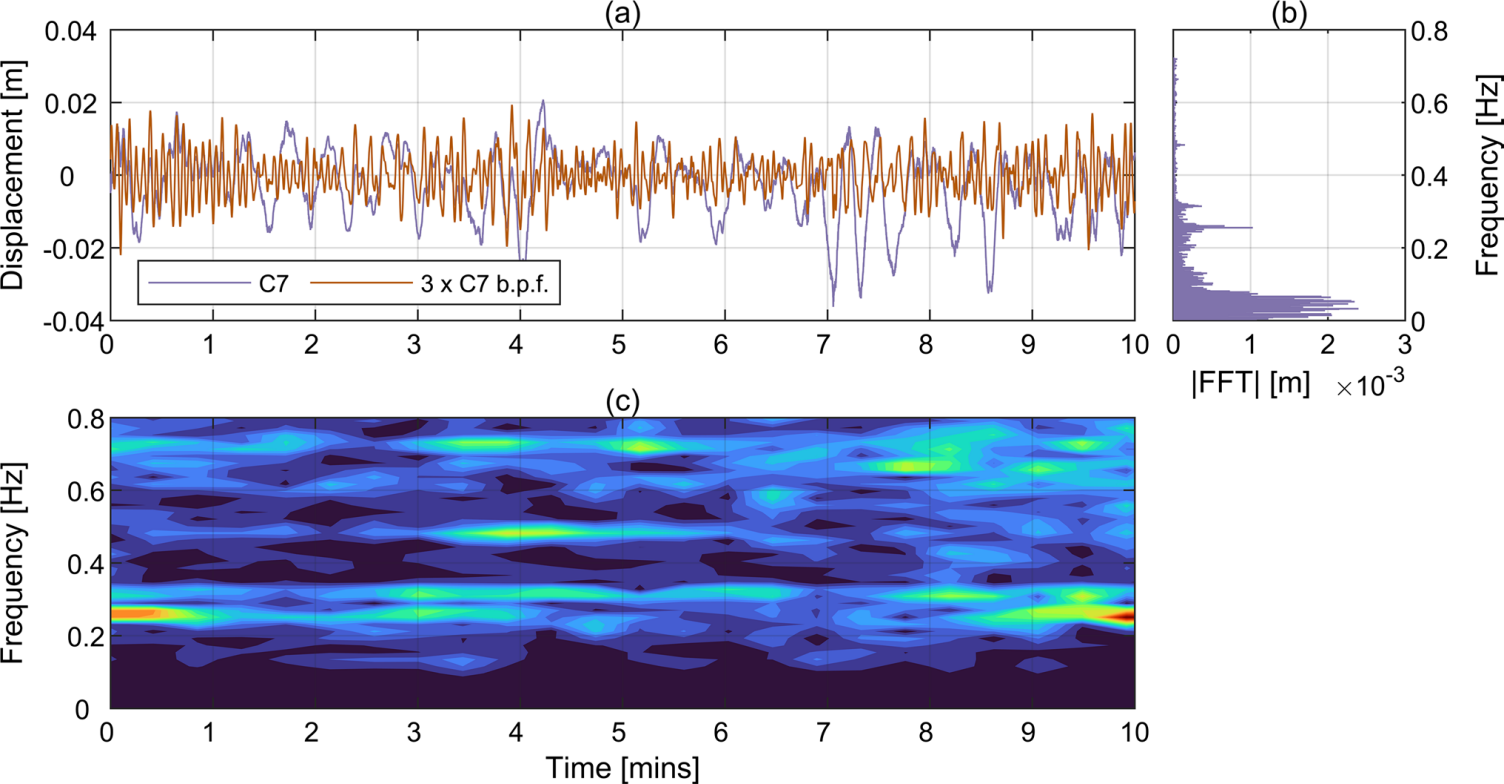
Displacement measured with CGC OF during T2 at C7. **a** Raw time history and time history after the application of band-pass filter within the range from 0.1 Hz to 0.9 Hz, **b** power-preserving FTF magnitude of raw displacement, **c** Fourier spectrogram of acceleration obtained by frequency scaling

**Fig. 8:**
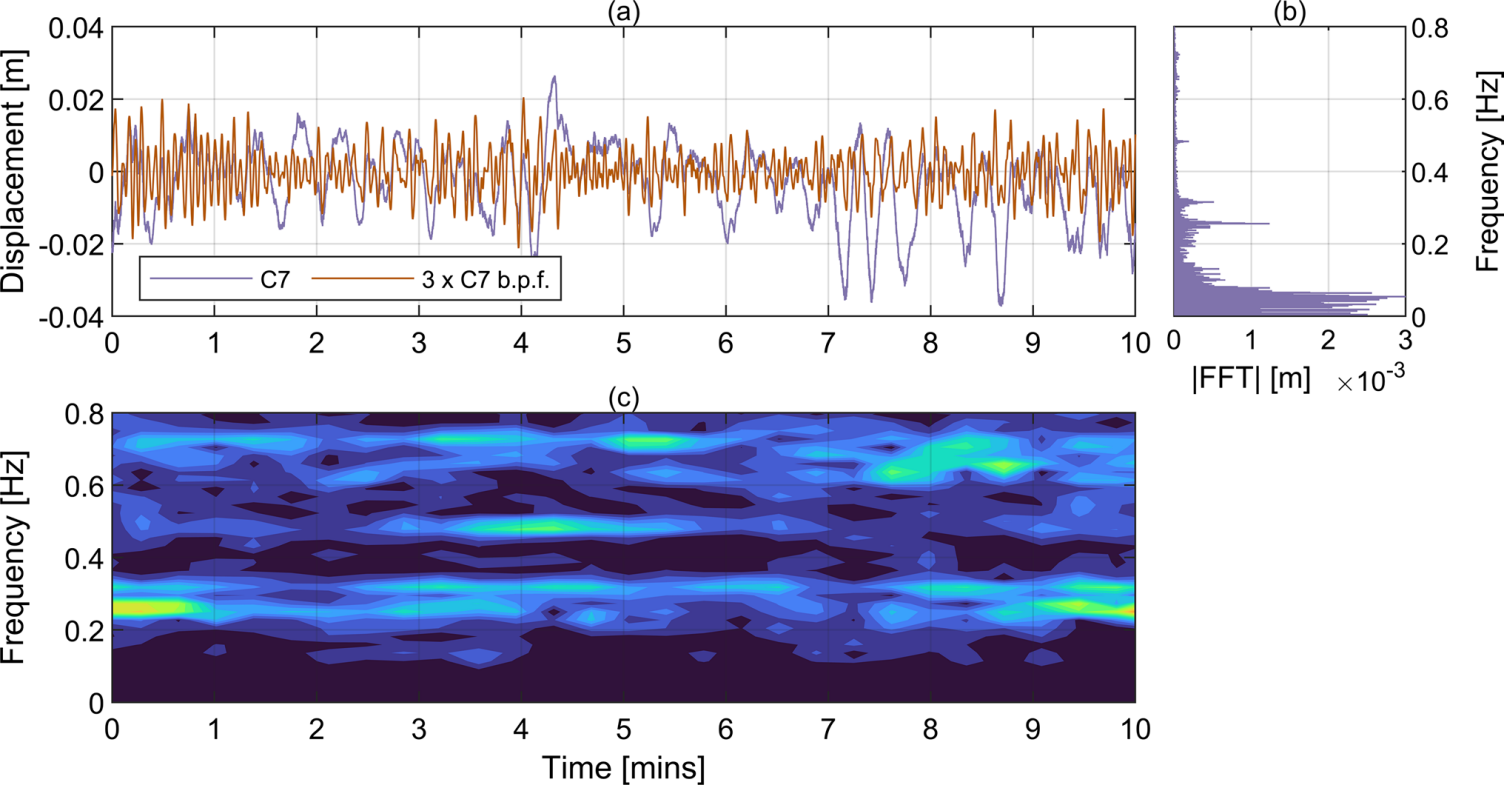
Displacement measured with Imetrum during T2 at C7. **a** Raw time history and time history after the application of band-pass filter within the range from 0.1 Hz to 0.9 Hz, **b** power-preserving FFT magnitude of raw displacement, **c** Fourier spectrogram of acceleration obtained by frequency scaling

For comparison, the corresponding data for T2 from CGC OF and Imetrum are shown in Fig. 7 and Fig. 8, respectively. Figure 7a presents the raw displacement measured with CGC OF at C7. The peak-to-peak amplitude is approximately 6 cm. The corresponding power-preserving magnitude of fast Fourier transform (FFT) is shown in Fig. 7b. The signal is dominated by the frequency components below 0.1 Hz, which are associated with the forced deflection of the bridge caused by the travelling vehicles. The amplitude of the frequency component associated with the first expected mode at approximately 0.26 Hz reaches 1 mm, but the amplitude for the fourth expected mode at approximately 0.62 Hz is below 0.05 mm. The displacement amplitudes for all frequency components within the range from 0.1 to 0.8 Hz comprise relatively little part of the total bridge’s response. To reveal this feature, the original signal was filtered with the 4^th^ order two-way Butterworth band-pass filter with cut off frequencies at 0.1 Hz and 0.9 Hz. The resultant signal, magnified threefold for better visibility, is shown in Fig. 7a. Figure7c presents a Fourier spectrogram of the acceleration obtained by scaling the band-pass filtered frequency-domain displacement signal by the squares of the angular frequencies. This was done to enable direct comparison against the acceleration spectrogram in Fig. 6c. Indeed, the same frequency components were excited intermittently in this case, indicating good correspondence with the directly measured acceleration. Figure 8 presents data obtained with Imetrum during T2 at C7. All plots are very similar to the corresponding plots for CGC OF in Fig. 7. The main difference is in the slightly higher motion amplitudes in Fig. 8a and Fig. 8b, which is most likely due to the different definition of the scaling factors adopted to convert readings from the camera to physical coordinate system, and less defined spectrogram in Fig. 8c, which can be due to the lower sampling frequency of Imetrum.

### Bridge response to the vehicular traffic

The main reason for the energy content below 0.1 Hz, as shown in Fig. 7b and Fig. 8b, is the loading from the vehicles travelling across the bridge. The cable-stayed continuous box girders, resting at the cross-beam within the pylon, are 512 m long between the first supports away from the pylon. For light vehicles, e.g. passenger cars, travelling at 120 km/h, it takes 15.36 s to cover that distance, corresponding to the frequency of 0.065 Hz. For heavy vehicles, e.g. lorries, travelling at 90 km/h, it takes 20.48 s to cover that distance, corresponding to the frequency of 0.049 Hz. The spread of speeds at which vehicles travel across the bridge explains the significant amplitudes of other energy components below 0.1 Hz.

**Fig. 9:**
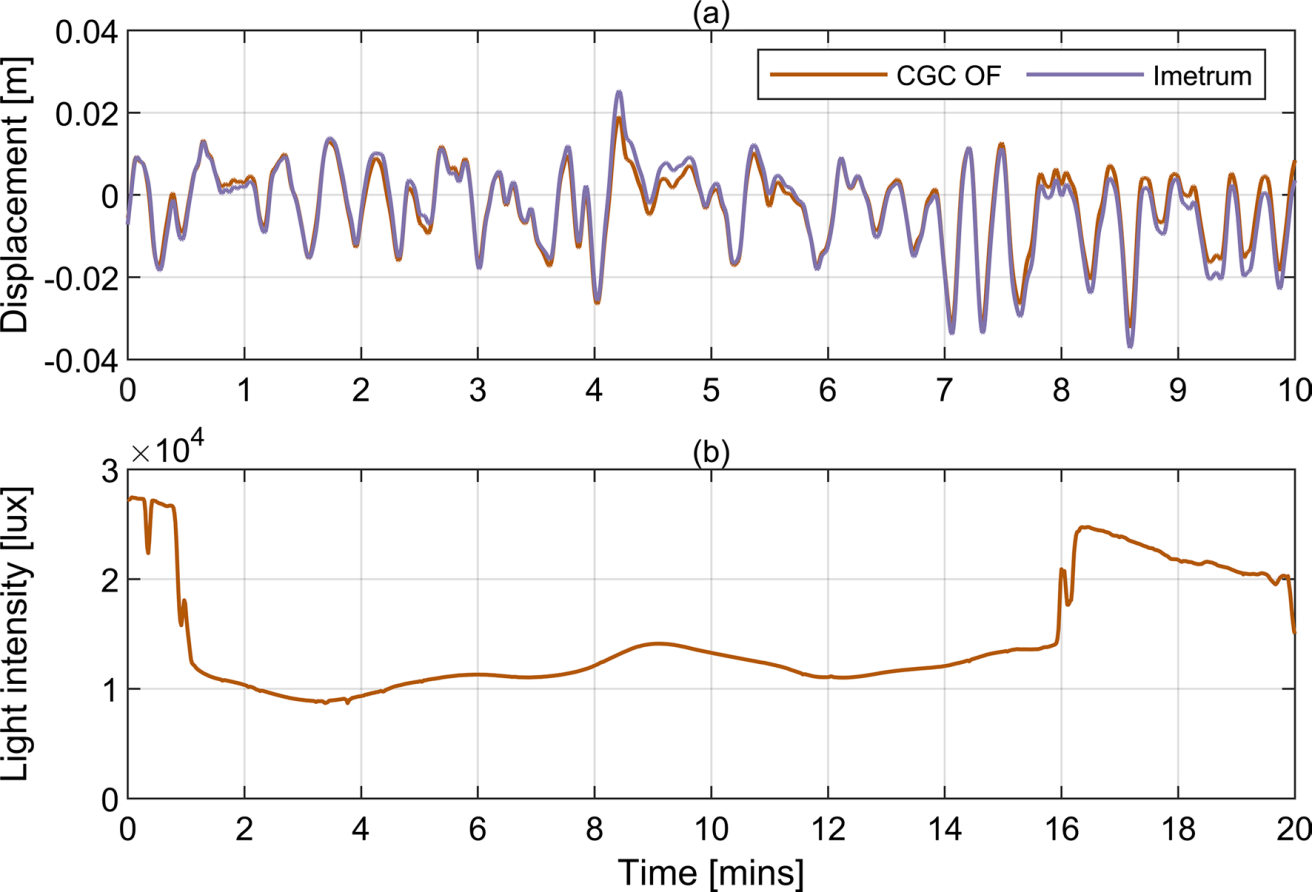
**a** Displacement of the Rędziński Bridge at C7 due to vehicular traffic according to signals from CGC OF and Imetrum collected during T2 and **b** light intensity measured during T2 at the GMS

The bridge response frequencies due to travelling vehicles fall well below the first modal frequency, which is generally a desirable characteristic from the point of view of bridge stability and fatigue as it avoids a resonant response. To evaluate the dynamic behaviour in this lowest frequency range, the outputs from optical MCS only were used, because they can capture data all the way down to the zero frequency component which is often not the case for accelerometers. Exemplar results obtained for CGC OF and Imetrum after applying a 4th order two-way Butterworth low-pass filter with cut off frequency at 0.2 Hz are shown in Fig. 9a. It can be seen that the match between signals is generally good, although slight differences in local amplitudes are sometimes present. The root-mean-square magnitudes of the signals are 0.0094 m and 0.0104 m for CGC OF and Imetrum, respectively.

To verify whether the less than 1 mm root-mean-square magnitude difference can be explained by the sensitivity of MCS to light intensity fluctuations, Pearson correlation coefficient was calculated between the measured signals and the data collected by the light meter shown in Fig. 9b. The time scale in Fig. 9b is incompatible with that in Fig. 9a because the two systems were operating independently hence uncertainty exists as to their time alignment. The level of illumination is typical of a cloudy day with some clear winter sky [39], which corresponds well to the conditions during the tests as shown in Fig. 4. The fluctuations in the light intensity are slow for the major part of the signal in Fig. 9b, but the signal drops and rises abruptly at around 1 min and 16 min.

The uncertainty in time alignment was accounted for by conservatively extending the time interval for the light intensity signal in which the correlation between data was examined and using a sliding window approach. The maximum correlation coefficient was –0.27. Low pass filtering the displacement signals had little influence on the results. Therefore, no clear relationship between the measured displacement due to vehicular traffic and light intensity was found. Therefore, it is likely that the slight difference in scaling from the image to physical units via homography matrices could explain some discrepancies between the results.

### Modal frequencies

**Fig. 10:**
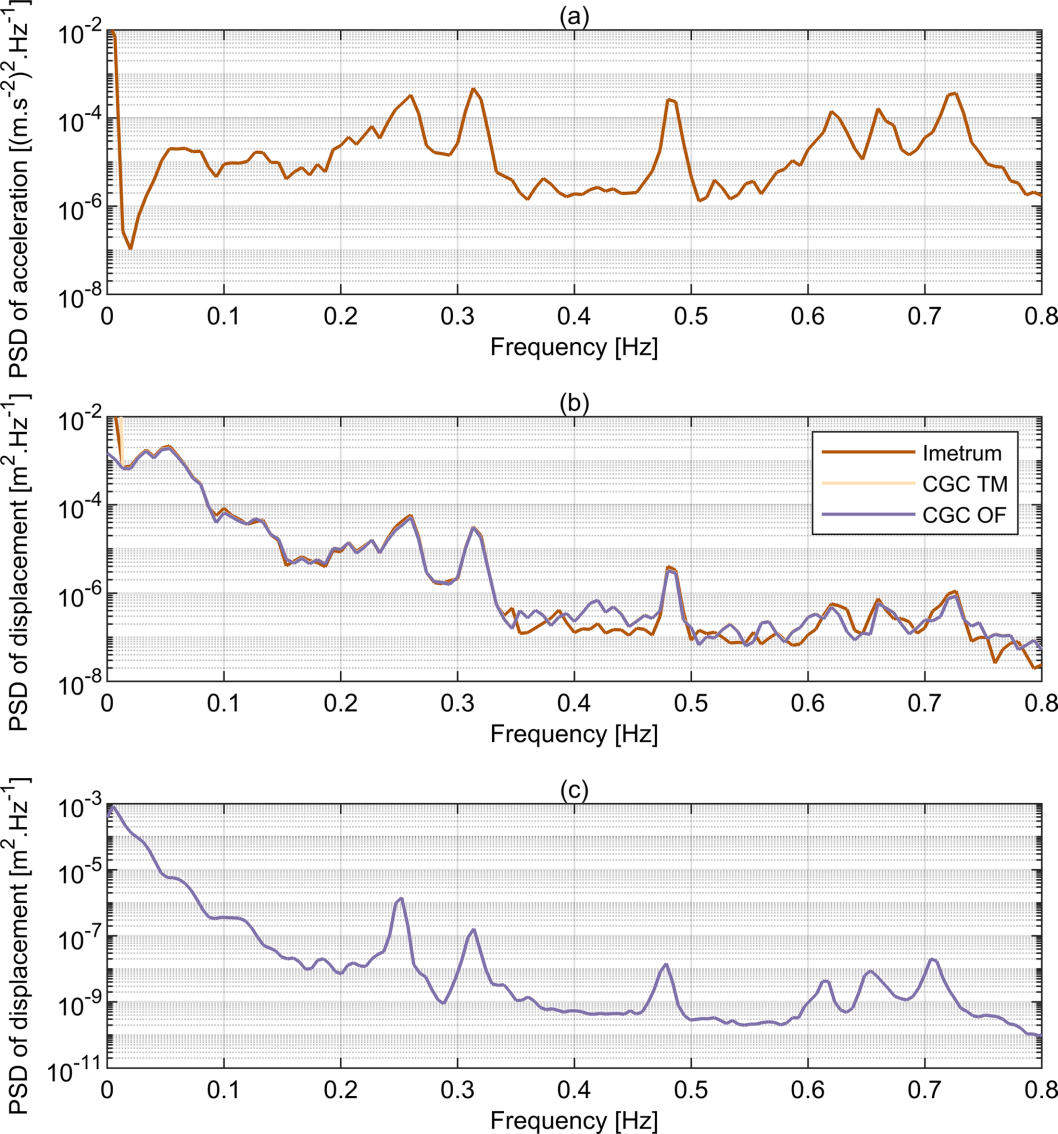
Power spectral densities obtained from data collected during T2 from **a** accelerometry, with frequency resolution of 0.0067 Hz, **b** Imetrum, CGC TM and CGC OF, with frequency resolution of 0.0133 Hz. **c** Power spectral density obtained from data collected during proof loading tests, with frequency resolution of 0.0051 Hz

The vibration frequencies obtained from MCS deployed in November 2020 were compared against those obtained from measurements taken during proof loading tests in August 2011. Exemplar power spectral densities (PSDs) of bridge response obtained from accelerometry and camera-based MCS during T2 are shown in Fig. 10a and b, respectively. The Welch method with 75 s Hanning window and 50% overlap was used based on signals of 10 min duration, giving frequency resolution of 0.0133 Hz. The six frequencies corresponding to the local maxima in the PSD magnitude were extracted, as seen in Fig. 10a, and the corresponding frequencies were then identified in the magnitudes of PSD of displacement, as seen in Fig. 10b. Figure 10c presents the PSD of displacement obtained from measurements taken from multiple lorry runs during the proof loading tests using the Welch method with approximately 194 s Hanning window and 50% overlap based on a signal of 52 min duration, giving frequency resolution of 0.0051 Hz. The response amplitudes at the peaks are much lower in this case than those in Fig. 10b and the peaks are shifted towards comparatively lower frequencies.

The frequencies identified from the current and previous measurements are shown in Table 3, together with mode shapes obtained from a numerical model [29]. In the case of the mode assigned number 3 in Table 3, a numerical model of the bridge actually predicts the existence of two closely spaced modes. However, neither set of measurements enabled this prediction to be verified, hence no distinction was made in this respect in further analysis and discussion of the results.

**Table 3:**
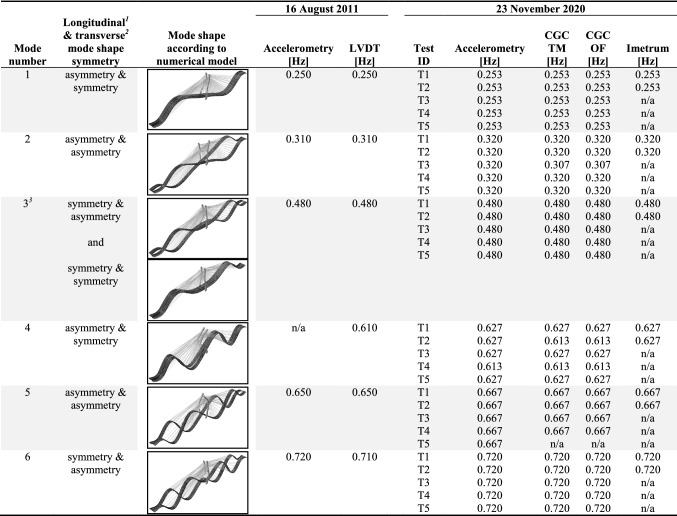
Comparison of the modal frequencies previously reported with current results

^1^Correlation in motion between the two sections of the cable-stayed span divided in half by the pylon

^2^Correlation in motion between the two independent parallel decks, each accommodating one-directional vehicular traffic

^3^Numerical model of the bridge predicts the existence of two closely-spaced modes [29]

Good stability of the results was achieved between the tests, indicating their length was sufficient to obtain reliable average values of modal frequencies. The difference between the results from various MCS and between various tests is never greater than the frequency resolution. The identified vibration frequencies correspond very well to those from previous measurements. The slight differences are predominantly caused by the temperature effects, hardening of concrete over time and various test durations and sampling frequencies as determined by the capabilities of the measurement systems stated in Table 1. The previous set of measurements with accelerometers was conducted on the 16th of August, 2011. The temperature on the shadowed and sun-exposed surfaces of the Rędziński Bridge during the testing day was measured at 19 to 21 and 19 to 32 degrees Celsius, respectively. The results reported in this study come from measurements conducted on the 23rd of November, 2020, with the temperature recorded by the nearest weather station between 6 and 8 degrees Celsius. The unavailability of results from CGC for mode 5 is caused by the low response levels for that mode during the proof loading tests.

### Modal damping

**Fig. 11:**
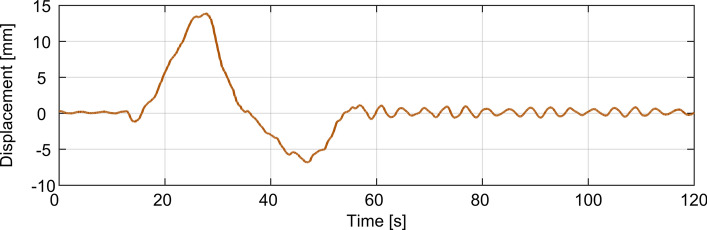
Bridge displacement at due to the passage of a lorry travelling at 50 km/h measured midway between C7 and C8

For the set of data collected in August 2011, modal damping was established based on tests D1 to D3, as defined in [26], during which one or three loaded lorries, each having mass over 40 tons, travelled across the bridge. An exemplar bridge response due to the passage of a lorry with mass 40.25 tons travelling at the speed of 50 km/h over a 3 cm high bump located within the middle lane of the south-east section of the deck carrying traffic towards Warsaw, as measured with LVDT positioned midway between C7 and C8 (the latter point not specifically denoted in Fig. 1 and Fig. 3), is shown in Fig. 11. The free decay response starts just before the 60^th^ second and it is preceded by the response under the moving lorry.

**Fig. 12:**
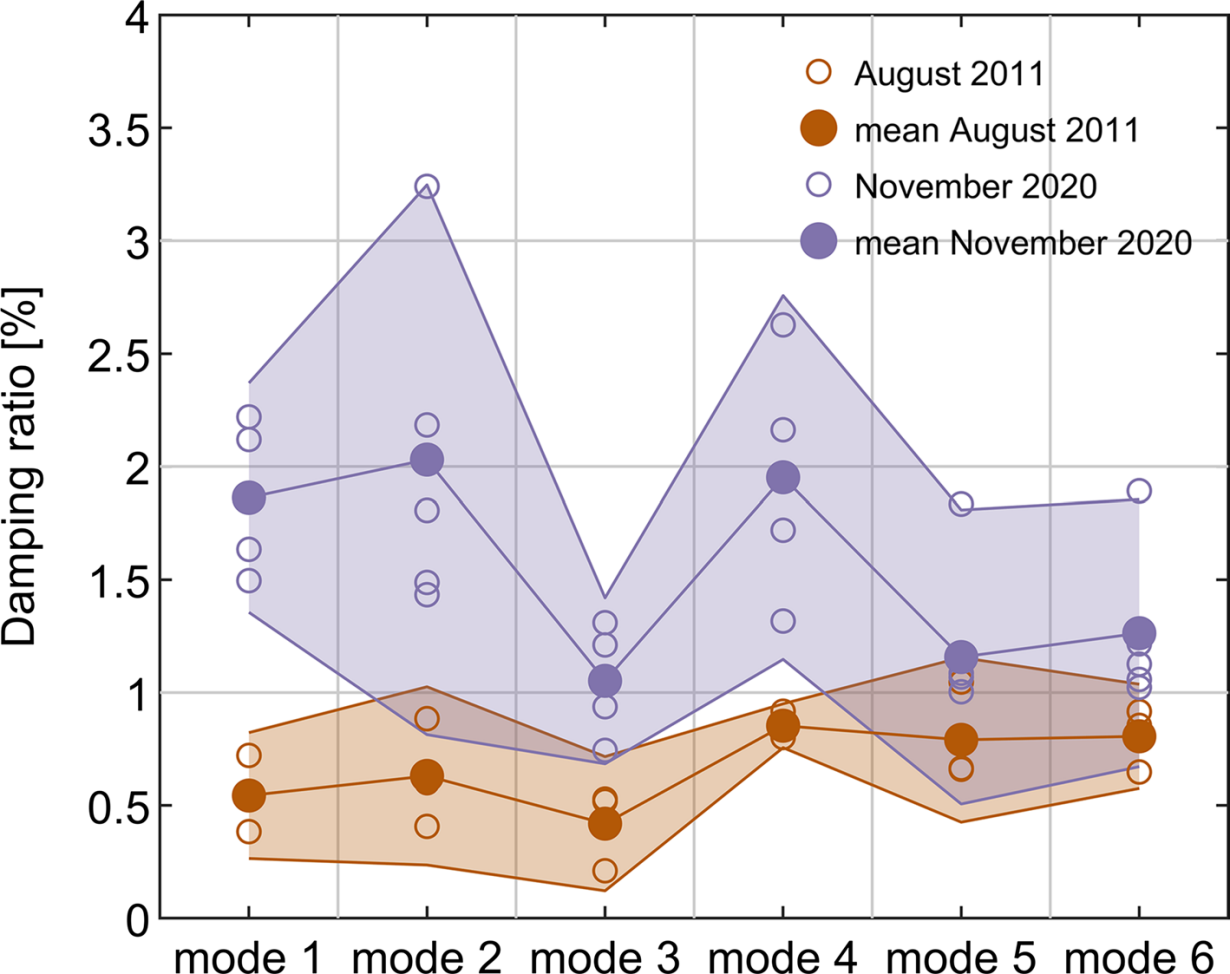
Damping ratio for the first six identified modes based on data collected in August 2011 from LVDT for tests D1 to D3, as described in [26], and data collected in November 2020 from accelerometry for tests T1 to T5. The values on the boundaries of the coloured patches for each mode denote 95% confidence intervals

The modal damping for modes 1 to 6, based on tests D1 to D3, was found to take values within the range of 0.2% to 1.05% of critical. Figure 12 depicts the mean damping ratio of each mode, also designating the associated 95% confidence intervals. The piecewise linear curves connecting the mean damping ratio values and 95% confidence bounds for consecutive modes have no physical significance per se, but they are presented as a means of highlighting trends from mode to mode. The extreme values of the 95% confidence bounds over all modes range from 0.1 to 1.2%. All the values falling within that range can be considered relatively low for a concrete-built construction. Still, overall, these damping ratios fall well within the range of values expected for large-scale bridges having similar modal frequencies to the Rędziński Bridge [40].

The mean damping ratios for modes 4, 5 and 6, denoted in Fig. 12 by the dots on the piecewise linear curve, seem to be relatively high, having almost identical values but quite disparate uncertainties, with mode 4 giving particularly consistent mean value between tests when compared to all other modes. Mode 3 is the lowest damped mode, with the damping ratio never exceeding 0.52% of critical.

The latest measurements from tests T1-T5, taken after over 9 years since D1-D3 and for which the ambient environmental conditions were very different, show a rather interesting picture.

It can be seen in Fig. 12 that the modal damping, as obtained from accelerometry, has generally increased significantly and consistently—considering the 95% confidence bounds, it now ranges from 0.8% to almost 3.2%. This increase is not uniform between modes, with modes 1 and 2 being affected the most and modes 5 and 6 the least.

This significant increase in overall damping of the Rędziński Bridge shares resemblance with findings from the latest long-term study on the evolution of modal damping for the Jindo Bridge, South Korea also supported on cable stays, reported in Hwang et al. (2021) [41]. Therein, there is a similar trend of damping increasing from summer to winter months (cf. Figure 13 in [41]), but at a level not comparable to the highest level reported herein, i.e. significantly less than twofold increase for the Jindo Bridge. Some previous studies have also reported the negative correlation between the modal damping and temperature [42, 43], although the interaction between the temperature and structural properties of large-scale bridges has received relatively little attention so far. The difference in temperature between the cable stays and the main concrete components of the Rędziński Bridge, i.e. pylon and decks, can reach 10 degrees Celsius [44]. Furthermore, the diurnal changes in temperature ranging from 20 to 40 degrees Celsius can cause tenfold larger changes in the tensile force than those associated with vehicle loading. The effect of differential temperature distribution on bridges requires in-depth, purpose-oriented investigations; monitoring practices like the ones reported herein are effectively enabling such novel lines of research.

**Fig. 13:**
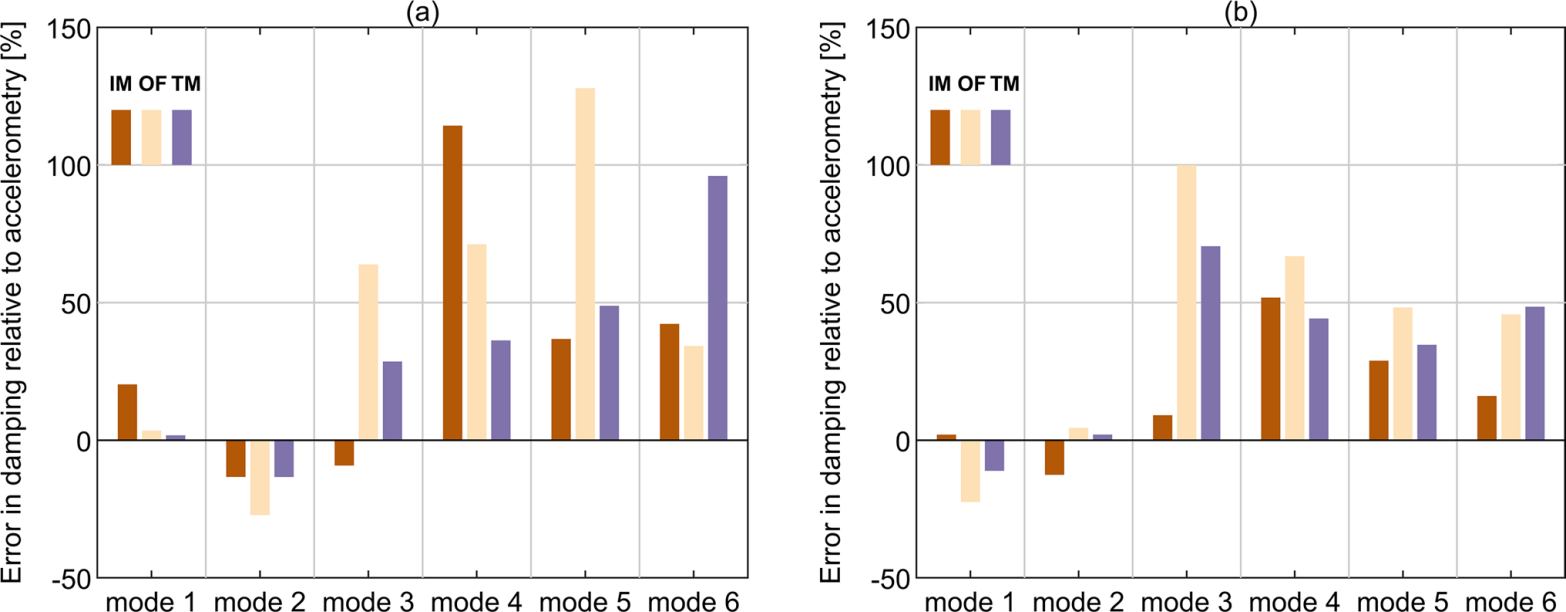
Error in damping relative to accelerometry based on data collected in November 2020 for **a** T2, and **b** the results averaged over all tests

Anticipated uncertainty bounds for modal damping as reported in [45] for a footbridge can lie within 15% to 30%, yet values identified herein prove to be higher on average. This is most likely caused by the different scale of the bridge considered herein and the relatively short data records used [46].

Mode 3 remains the lowest damped mode, with a mean value around 1% of critical, but now modes 1, 2 and 4 are the most damped with mean values around 2% of critical. Unfortunately, decoupling the influence of different parameters on damping evolution is impossible with the limited data set available. However, the current study is believed to be one of the few works capturing the influence of the combined long-term and seasonality effects on modal damping for long-span bridges, showcasing their significance and potential magnitude. Among others, this is sine-qua-non towards employing damping as an index in damage detection studies.

Having identified the baseline values of modal damping, it is now possible to compare the performance of all remote monitoring systems applied in this study, as in the case of laboratory-based work presented in [8, 9]. Figure 13a illustrates the identified modal damping error against accelerometry for Imetrum and CGC systems during the exemplar test T2. Although only OF data were presented when discussing the results in previous sections owing to the seemingly insignificant deviations between OF and TM, results obtained with both methods are now shown. This is because the small differences in the bridge response between OF and TM translate into much more pronounced discrepancies in modal damping. For mode 1, both the OF and TM perform better than Imetrum, with relative errors for the three systems of 3.51%, 1.79% and 20.21%, respectively, with the sign indicating that all the camera-based systems overestimated damping in this case. For mode 2, the corresponding errors are  – 27.18%, – 13.35% and – 13.33%, even though the typical picture observed is optical systems yielding higher damping values for the identified modes. Therefore, overall, the performance of optical MCS for modes 1 and 2 is not far from accelerometry, particularly when considering the confidence intervals denoted in Fig. 12. For higher modes, but apart from mode 6, the errors grow almost monotonically to as high as 127.9%, 96.03% and 114.29% for OF, TM and Imetrum, respectively. It is concluded based on T2 only, that, at least for the purpose of damping identification, TM performs better than OF for all modes apart from mode 6, where OF shows the best performance among all considered optical systems. This finding is not fully in-sync with the findings in [9] where the equivalent performance during forced Input–Output (IO) tests was much closer between OF and TM, with a slight advantage of OF over TM. In general, this could be sign of a greater immunity of TM to ambient full-scale conditions and gives merit to using both TM and OF techniques, particularly for damping identification purposes. Figure 13b shows the average error in modal damping relative to the results obtained from accelerometry over all available tests (i.e. T1-T5 for OF and TM, and T1 & T2 for Imetrum). Although the errors for modes 1 and 2 for CGC-based systems change sign, they are still small. However, the trend in the errors magnitude for modes 3 to 5 for these systems reverses, i.e. the errors decrease from mode 3 to 5. This is likely due to the various levels of excitation of the modes during individual tests and the effect of vehicles crossing the bridge, which can add modal damping. Their performance seems consistent and reliable with extreme deviations falling significantly over the 95% confidence intervals denoted in Fig. 12 only in the case of mode 3. This is the least damped mode in any of the tested cases and, as can be seen in Fig. 6, it is excited stronger than modes 5 and 6. Therefore, this discrepancy cannot be attributed to amplitude-related effects.

**Fig. 14:**
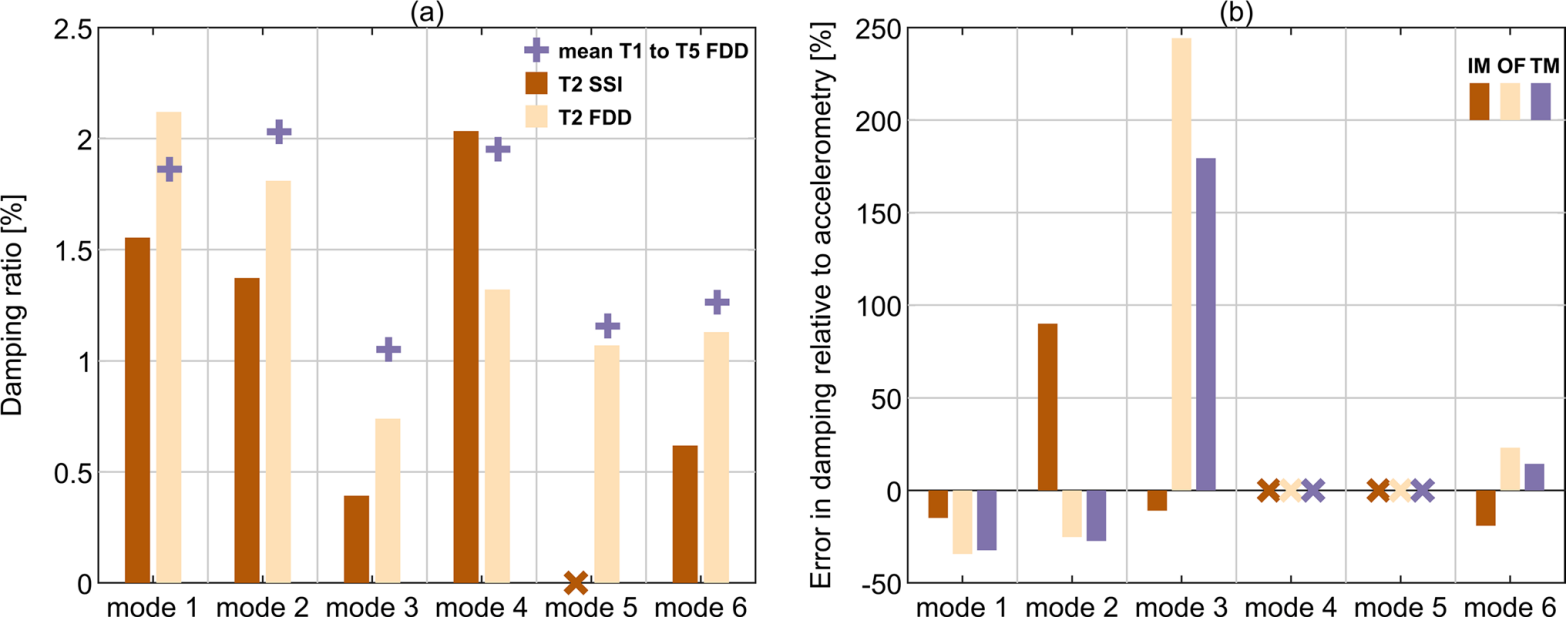
**a** Damping ratio obtained using SSI and FDD for accelerometry for T2. The mean damping ratio over all tests obtained with FDD is also included. **b** Error in damping ratio relative to accelerometry for all camera-based MCS obtained with SSI for T2

As explained in Sect. 2.4, FDD was employed for acquiring all the damping properties previously discussed. Applying SSI towards identifying damping was also tested in order to waive any manual handling artefact and gain confidence in that the relationships that could be seen in the derived modal damping are immune to the damping extraction method. Figure 14a presents damping ratio obtained using FDD and SSI for T2, and the average damping ratio over tests T1 to T5 obtained using FDD. Figure 14b presents the error in modal damping relative to the results from accelerometry using SSI for the exemplar test T2.

Considering the case of T2 only, SSI yields damping ratio lower by 30% to 80% relative to the results from FDD. The only exception is mode 4, for which the bridge response was of low amplitude. In this case, SSI yields a higher damping ratio estimate relative to FDD, yet closer to the average value of the damping ratio over all five tests obtained with FDD. For mode 5, SSI failed to identify a stable mode during the identification process.

Considering the errors in modal damping obtained from data collected with all camera-based MCS using SSI relative to the results from accelerometry, the best results were found for modes 1, 2 and 6. The error magnitude is there always below ± 35%, except the error for Imetrum for mode 2 which is slightly above 90%. Therefore, apart from this exception, the errors fall within the uncertainty bounds presented in Fig. 12. The error in modal damping increased significantly for mode 3, which has the lowest damping of all modes. This may seem unacceptable at the first glance, but the actual damping ratios obtained using SSI for TM and OF at 1.098% and 1.353%, respectively, are close to the average value over tests T1 to T5 obtained using FDD from accelerometry, at 1.052%. It seems that the error increase should mainly be attributed in this case to the lower modal damping ratio for T2 as can be seen in Fig. 14a. Modes 4 and 5, which were the least excited during T2, as shown in Fig. 6 and Fig. 10, do not yield stable modes and as such damping results for comparison.

The advantage of TM over OF identified in the results obtained with FDD is still preserved in SSI, but it is less pronounced.

To conclude, the identified damping ratios are not out of context and clearly there is a complex interaction of data source, the mode specifics and damping extraction method.

**Fig. 15:**
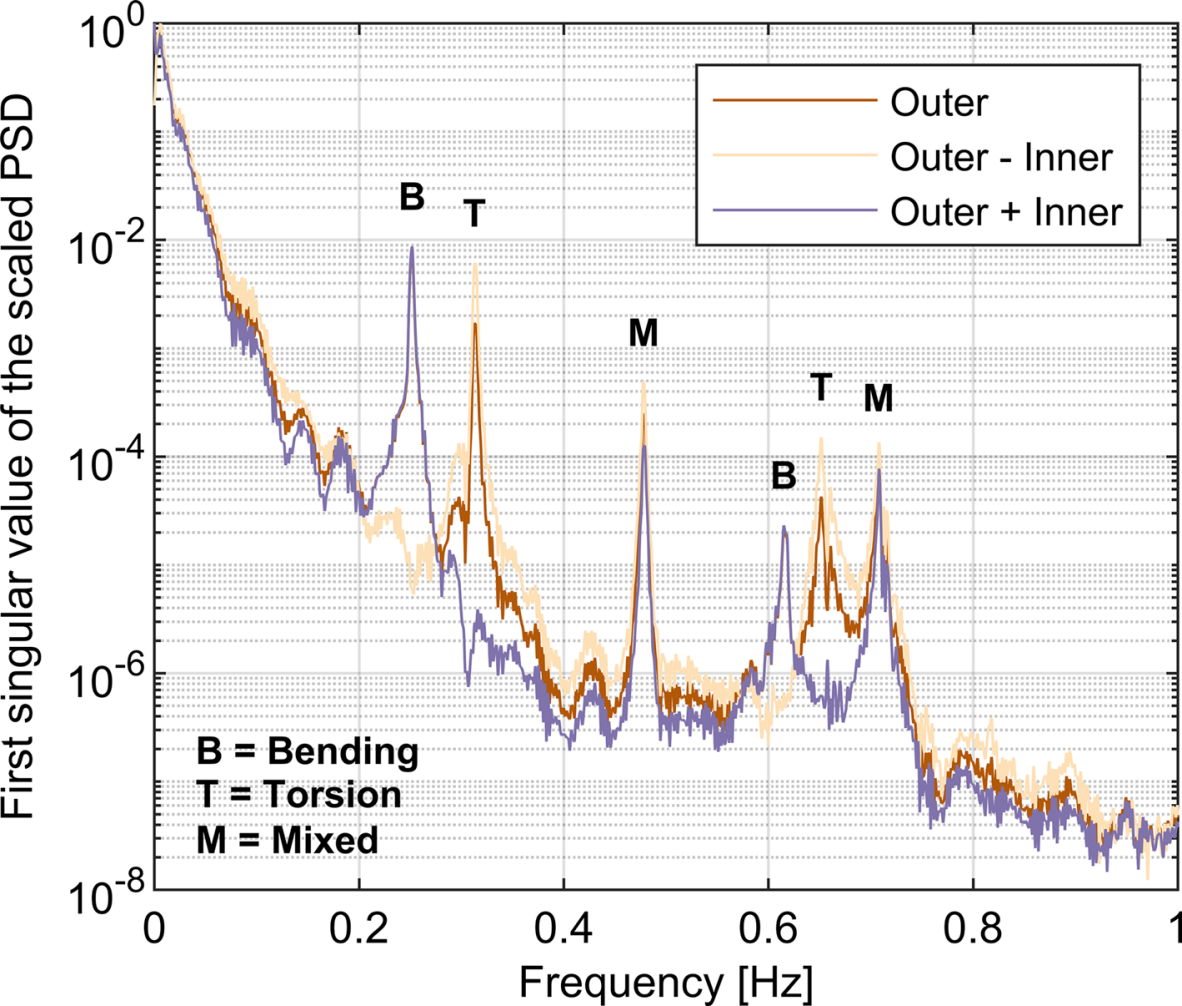
Scaled power spectral density (PSD) of the Rędziński Bridge response from data collected in August 2011 used to evaluate the nature of each mode

In general, it is impossible to distinguish, solely relying on the measurements taken with camera-based MCS in November 2020, whether identified modes are of bending, torsional or mixed nature. However, it may be conjectured that this characteristic can affect the damping identification process and impact the results [8]. To clarify this issue, Fig. 15 presents power spectral densities (PSDs) from bridge response data collected in August 2011 during D1 tests. A dataset consisting of signals from two instruments positioned symmetrically relative to the longitudinal axis of a single deck at the inner (i.e. closer to the other deck) and outer (i.e. further away from the other deck) edge was used. These signals are denoted within Fig. 15 simply as “inner” and “outer”. In simple terms, the half-difference of the signals from the two instruments serves to decouple the torsional motion about the midpoint, that falling onto the longitudinal axis of the deck, while their summation yields a multiple of the remainder. As can be seen in Fig. 15, modes 1 and 2, for which damping derived from the optical MCS closely matches that from accelerometry, are of bending and torsional nature, respectively. Mode 3, for which there is considerable uncertainty in damping identification, is of mixed nature, yet mode 6, which is also of mixed nature, seems well identified. Modes 4 and 5 are of bending and torsional nature, respectively. In view of this, current findings do not support the aforementioned conjecture. Therefore, this issue requires further investigation.

It needs to be pointed out on this opportunity, that the earlier work on the Rędziński Bridge (cf. Table 3 in [26]) did not clearly distinguish torsion within the identified mode shapes and as such this study not only completes but also corrects previously published results for the Rędziński Bridge.

## Conclusions

To the best of the authors’ knowledge, this is one of the very first and most methodical attempts to showcase and validate the applicability of camera-based vibration monitoring systems in the determination of modal damping from large-scale bridges in situ. It is also unique in reporting observations of how modal damping evolves under the combination of long-term and seasonal effects in the course of approximately 9 years of operation.

Overall, despite their remote sensing nature and inherent vulnerability to the environmental conditions (e.g. light intensity fluctuations and refraction), the camera-based MCS (i.e. two systems based on CGC and Imetrum) proved capable of providing high-quality data on the structural response. For the particular application presented herein, CGC turned out more reliable than Imetrum in terms of the ease of setting up and robustness against image overexposure. All camera-based MCS were able to capture data all the way down to the DC level (i.e. zero frequency component), which is a desirable characteristic for static and quasi-static load tests, e.g. incremental loading.

The dynamic behaviour of the Rędziński Bridge in normal operational conditions, representative of those during a short experimental campaign in November 2020, is dominated by the response to vehicular traffic. This response is associated with the spectral components below 0.15 Hz. The dynamic effects of vehicular loading are well separated from the modal properties of the bridge with the six lowest modal frequencies occurring between approximately 0.26 Hz and 0.72 Hz. Despite small amplitude dynamic bridge response in these modes, the quality of the captured data was found sufficient for the modal damping to be determined using any approach, although uncertainty bounds were found seemingly wide.

The mean damping ratio established from data collected during the commissioning campaign of the Rędziński Bridge undertaken in August 2011 was calculated for the first time and found to be rather low for concrete-based structure, with values within the range of 0.42% to 0.85%. The corresponding range of damping ratio established from data from accelerometry collected in November 2020 is 1.05% to 2.03%. This large increase in the mean damping ratio is believed to be mainly caused by the combination of two factors, namely changes in materials’ properties over time and the significant difference in ambient temperature between the two experimental campaigns. The effect of damping increase is spread non-uniformly among modes with the lowest two modes showing the strongest variation. The uncertainty bounds for the damping ratio are wider for the more recent set of data, which is believed to be caused by the non-canonical levels of vehicular traffic on the bridge between all short tests.

The absolute error in mean modal damping derived using frequency-domain decomposition (FDD) from data obtained with camera-based motion capture systems in November 2020, relative to the results from accelerometry, falls below 23% and 13%, respectively, for mode 1 and 2. The corresponding error for results derived using stochastic subspace identification (SSI) is 35% and 28%, excluding the results from Imetrum for mode 2. The results from consumer-grade camera (CGC) for the two lowest modes compare favourably with the typical uncertainty in damping identification reported from accelerometry for large-scale bridges. The results for higher modes are less reliable, with the absolute error reaching almost 100% in the worst case. This is predominantly caused by the low excitation of these modes. For the exemplar test (T2), modal amplitudes for modes 1 to 3 were, respectively, 1.0, 0.3 and 0.2 mm, and well below 0.1 mm for the rest three considered modes.

The template matching algorithm (TM) outperformed optical flow algorithm (OF) in most cases and provided data of similar quality to Imetrum at a fraction of the cost.

Overall, the results presented in this study could act as a go-to reference encouraging further efforts in exploring the application of camera-based motion capture systems (MCS) towards the identification of dynamic behaviour of large-scale bridges. Such practices could be particularly useful for structures where no permanent structural health monitoring system is installed, as is often the case for older bridges. Provided the mode of interest is sufficiently excited, these systems can provide reliable information on modal damping and unleash great opportunities in research towards better understanding and mapping the evolving patterns of dynamic behaviour in ageing structures, potentially serving in damage detection.
